# Case of Extirpation of the Eye

**Published:** 1839-09-21

**Authors:** George M‘Clellan


### CASE OF EXTIRPATION OF THE EYE.

By George M'Clellan, M.D.

[Reported by Dr. B. J. Blankman.]

This case was a beautiful little girl, about two years old. The eye had been protruding for ten weeks, and finally it became an immense exopthalmus. Vision had not declined in it until eight or ten days before Dr. M'Clellan saw it. But the external tunics had then become greatly enlarged, and painful irritation had supervened. It was difficult to understand what the cause of protrusion could be, for a medullary growth upon the optic nerve would have destroyed the vision in that eye at first. There was considerable constitutional irritation present previous to the operation; the child was restless, and the eye apparently very painful, and continuing to grow worse every day, until the eye was extirpated. The operation was begun, first, by enlarging the outer angle of the lids by an incision outwards, and then plunging the knife behind the ball. A prodigious hæmorrhage of black blood gushed out from this wound. The next step to the operation was to remove the globe, by plunging a bistoury behind the inner canthus of the lids, and cutting off the muscular and nervous attachments between the ball and the foramen opticum. A large mass of discoloured cellular tissue was then exposed at the bottom of the orbit, from which a tremendous hæmorrhage issued. As soon as this was removed, the hæmorrhage ceased, and on examining it afterwards, it was found to contain several vascular cysts, into which the blood had been exhaled, and from the cavity of which the hæmorrhage had no doubt escaped. They were evidently blood cysts, such as Mr. Craig has denominated "hematomes." On cutting open the ball, it was found that the black pigment had nearly disappeared from the back part of the eye, and the choroid presented a reddish hue. The little girl is doing well. This was certainly different from the cases of aneurism by anastimosis.

The recovery has continued perfect unto the the present date.

*September* 21.

